# Community Metabolic Interactions, Vitamin Production and Prebiotic Potential of Medicinal Herbs Used for Immunomodulation

**DOI:** 10.3389/fgene.2021.584197

**Published:** 2021-02-03

**Authors:** Christine T. Peterson, Stanislav N. Iablokov, Sasha Uchitel, Deepak Chopra, Josue Perez-Santiago, Dmitry A. Rodionov, Scott N. Peterson

**Affiliations:** ^1^Department of Family Medicine and Public Health, Center of Excellence for Research and Training in Integrative Health, School of Medicine, University of California, San Diego, La Jolla, CA, United States; ^2^A.A. Kharkevich Institute for Information Transmission Problems, Russian Academy of Sciences, Moscow, Russia; ^3^Department of Theoretical Physics, P.G. Demidov Yaroslavl State University, Yaroslavl, Russia; ^4^Department of Biology, Washington University, St. Louis, MO, United States; ^5^Department of Ayurveda and Yoga Research, Chopra Foundation, Carlsbad, CA, United States; ^6^Puerto Rico Omic Center Genomics Core Division of Cancer Biology, University of Puerto Rico Comprehensive Cancer Center, San Juan, Puerto Rico; ^7^Bioinformatics and Structural Biology Program, Sanford Burnham Prebys Medical Discovery Institute, La Jolla, CA, United States

**Keywords:** prebiotic, microbiota, immunomodulation, syntropy, short chain fatty acid, vitamin, metabolism, Ayurveda

## Abstract

Historically, the health benefits and immunomodulatory potential of medicinal herbs have been considered an intrinsic quality of the herb itself. We have hypothesized that the health benefits of medicinal herbs may be partially due to their prebiotic potential that alter gut microbiota leading to changes in short chain fatty acids and vitamin production or biotransformation of herb encoded molecules and secondary metabolites. Accumulating studies emphasize the relationship between the gut microbiota and host immune function. While largely unknown, these interactions are mediated by secreted microbial products that activate or repress a variety of immune cell types. Here we evaluated the effect of immunomodulatory, medicinal Ayurvedic herbs on gut microbiota *in vitro* using 16S rRNA sequencing to assess changes in community composition and functional potential. All immunomodulatory herbs displayed substantial prebiotic potential, targeting unique taxonomic groups. Application of genome reconstruction and analysis of biosynthetic capacity of herb selected communities suggests that many of the 11 herbs tested altered the community metabolism as the result of differential glycan harvest and sugar utilization and secreted products including multiple vitamins, butyrate, and propionate that may impact host physiology and immune function. Taken together, these results provide a useful framework for the further evaluation of these immunomodulatory herbs *in vivo* to maintain immune homeostasis or achieve desired regulation of immune components in the context of disease.

## Introduction

Ayurveda, the traditional system of medicine in India, emphasizes gastrointestinal health with medicinal herbs, dietary, and lifestyle medicine. Herbal medicines used for immunomodulation in Integrative and Ayurvedic Medicine were the focus of the current study (Table 1), however many medical herbs indicated for immunomodulation have been studied in this context and thus the current list is not exhaustive (Peterson et al., 2017, 2018, 2019b). These herbal medicines are also indicated for several other targeted therapeutic uses such as gastrointestinal and cardiovascular health (Poojari, 2014; Amalraj and Gopi, 2017). Several plant-derived constituents, such as antioxidants, polysaccharides, terpenoids, and flavonoids, are known to modulate immune status as well as the gut microbiota structure and function (Ichikawa and Aggarwal, 2006; Kumar et al., 2012; Peterson et al., 2017; Tiwari et al., 2018; Dai et al., 2019). For example, andrographolide is a diterpenoid of *Andrographis paniculata* (common name: kalmegh) that has been associated with reduced symptoms of upper respiratory tract infections in human clinical trials and mouse studies (Poolsup et al., 2004; Saxena et al., 2010; Tan et al., 2016).

**TABLE 1:** Herbal medicines examined in the current study.

**Common Name**	**Species**	**Family**
Arjuna	*Terminalia arjuna*	Combretaceae
Hawthorn berry	*Crataegus laevigata*	Rosaceae
Kalmegh	*Andrographis paniculata*	Acanthaceae
Kutki	*Picrorhiza kurroa*	Plantaginaceae
Manjistha	*Rubia cordifolia*	Rubiaceae
Musta	*Cyperus rotundus*	Cyperaceae
Punarnava	*Boerhavia diffusa*	Nyctaginaceae
Vidanga	*Embelia ribes*	Primulaceae
Vidari kanda	*Ipomoea digitata*	Convolvulaceae
Kanchanar guggulu	*Commiphora mukul*	Burseraceae
	*Bauhinia variegata*	Fabaceae
	*Terminalia chebula*	Combretaceae
	*Terminalia bellirica*	Combretaceae
	*Emblica officinalis*	Phyllanthaceae
	*Zingiber officinale*	Zingiberaceae
	*Piper nigrum*	Piperaceae
	*Piper longum*	Piperaceae
	*Crateva nurvala*	Capparaceae
	*Cinnamomum verum*	Lauraceae
	*Cinnamomum tamala*	Lauraceae
	*Elettaria cardamomum*	Zingiberaceae
Triphala guggulu	*Commiphora mukul*	Burseraceae
	*Terminalia chebula*	Combretaceae
	*Terminalia bellirica*	Combretaceae
	*Emblica officinalis*	Phyllanthaceae
	*Piper longum*	Piperaceae

*Selected common names and family information are shown.*

In the current study, we used anaerobic fecal cultivation to evaluate the modulatory potential of medicinal herbs on community composition by sequencing the 16S rRNA gene region V3–V4. The supplementation of a chemically defined media lacking exogenous carbohydrate sources with medicinal herbs had profound effects on the bacterial taxa observed indicating that constituents of each herb are actively metabolized by gut microbiota leading to changes in community metabolism. The concept of community metabolism is unique to microbiomes that exhibit numerous cooperative interactions to increase the fitness of taxa in a substrate dependent manner. While some of the effects of medicinal herbs are direct, other alterations may be the result of cross feeding relationships wherein the metabolic products of primary consumers are utilized by additional taxa that also gain a fitness advantage.

The study of the modulatory effects of medicinal herbs *in vitro* has the important advantage by permitting the pure microbiological effects of medicinal herbs in the absence of complicating host-dependent effects. Ultimately these *in vitro* effects serve as an important vehicle for understanding how microbes utilize herb substrates to provide a rational basis for selecting medicinal herbs that may be evaluated in human subjects.

Here, we make extensive use of genome reconstruction to predict the metabolic capacity of herb-selected gut microbiota. This approach is powerful and extends the utility of 16S rRNA sequence data to enable functional analyses in the form of predicted phenotypes pertaining to vitamin, SCFA and amino acid biosynthesis, glycosyl hydrolase functions and sugar utilization of complex microbial communities (Leyn et al., 2017; Romine et al., 2017; Feng et al., 2020).

The results presented indicate that each medicinal herb investigated alters gut microbiota uniquely exerting distinct positive and negative selection to a wide variety of bacterial species. Consistent with the unique selective pressure presented by each medicinal herb, community metabolism impacting vitamin biosynthesis and SCFA production were observed. Mechanistically, analysis of glycosyl hydrolase gene abundance and sugar utilization pathways encoded by herb-selected fecal microbiomes suggest that degradation of complex carbohydrates and subsequent sugar metabolism represent an important driving force of the observed modulatory capacity of each medicinal herb.

Taken together, the results support the possibility that the therapeutic benefits generated by immune modulatory medicinal herbs may be due in part to their ability to alter community metabolism of gut microbiota. This idea was recently demonstrated by feeding two prebiotic compounds, inulin or mucin to mice that enhanced their ability to mount effective anti-tumor responses and attenuate tumor growth (Li et al., 2020). In this regard, the potential of immune stimulating medicinal herbs to prevent and/or treat a wide variety of diseases represent an exciting area for future exploration. Thus, the authors hypothesized that the substrates present in herbal medicines may alter the gut microbiota composition and vitamin production thereby redirecting community metabolism.

## Materials and Methods

### Study Participants and Sample Collection

Healthy, English-speaking women and men aged 30–60 years that had previously adhered to a vegetarian or vegan diet for >1 year were recruited to donate a single stool sample. The study was approved by the Sanford Burnham Prebys Medical Discovery Institute Institutional Review Board (IRB-2014-020) and all study participants provided written informed consent prior to participation. Participants ate their normal diets and donated a morning fecal sample in stool hats (Fisher Scientific) that were subsequently placed on ice. The fecal samples were transferred to conical tubes and stored at −80°C until further processing.

### Medicinal Herbs Examined in the Current Microbiome Study

We examined 11 medicinal herbs in this study (Table 1). Organically grown herbal medicine powders were sourced from Banyan Botanicals (Albuquerque, NM, United States).

#### Anaerobic Fecal Cultures

Equal volumes of stool collected from 12 healthy vegetarian participants were pooled and used to inoculate [approximately 10 (Tiwari et al., 2018) cells] a chemically defined medium (CDM) supplemented with 1% herb (w/v). Anaerobic cultures (9% H_2_, 81% N_2_) were grown statically for 2–3 days at 37 C as technical replicates (*n* = 6) and grown to approximate saturation.

#### Chemically Defined Medium

Chemically defined medium contains 50 mM HEPES, 2.2 mM KH_2_PO_4_, 10 mM Na_2_HPO_4_, 60 mM NaHCO_3_, 4 mM of each amino acid, except leucine (15 mM), 10 mL ATCC, Trace Mineral Supplement. CDM contained nucleoside bases (100 mg/L), inosine, xanthine, adenine, guanine, cytosine, thymidine, and uracil (400 mg/L). CDM contained choline (100 mg/L), ascorbic acid (500 mg/L), lipoic acid (2 mg/L), hemin (1.2 mg/L), and myo-inositol (400 mg/L). Resazurin (1 mg/L) was added to visually monitor dissolved oxygen. The pH of the media was adjusted to 7.4. The 2× CDM and medicinal herbs (powder) in sterile water (2%) were separately reduced in an anaerobic chamber (Coy Labs) for 3 days.

#### Microbial DNA Isolation

Genomic DNA was isolated from cultures as well as the fecal inoculum using the procedures of the QiaAmp DNA stool kit (Qiagen) with a modification that included an additional step of bead beating with zirconia beads (100 micron) for 5 min using the Thermo FastPrep instrument (MP Bio) to ensure uniform lysis of bacterial cells.

#### 16S rDNA Sequence Analysis

Multiplexed 16S rDNA libraries were prepared using standard 16S metagenomic sequencing library protocols from Illumina, which uses V3–V4 region of 16S rDNA for target amplification. We performed paired end reads (250 bp) sequencing to generate ∼200,000 sequences/sample using the Illumina MiSeq. Subsequent analysis was done in CLC Microbial Genomics Module 2.5 (Qiagen) and R (Eglen, 2009). Paired end reads were merged (mismatch cost–2, minimum score–8, gap cost–3, maximum unaligned end mismatches–0) and trimmed to the same length. Additional quality filter steps were applied to exclude short reads, sequences with poor quality scores, and chimeras. To ensure comparable high coverage in all samples, we excluded samples producing <5,000 high quality reads. The average number of reads after quality filtering was 140,273 (range = 53,161–324,126) representing an average of 83% of the raw reads generated (range = 76–92%). The quality filtered reads were normalized to allow comparison across cultures. We did not use OTU-based enumeration of taxa due to the over-merging that occurs. Instead each unique 16S rDNA sequence was subjected to BLAST using the NCBI 16S rRNA database (Bacteria and Archaea) to identify best matches to taxa at the genus and species levels. The Principal Coordinates Analysis (PCoA) was generated using a multiple alignment of 16S amplicon sequence variants (ASVs) with MUSCLE. The resulting alignment was used to build an unrooted tree with FastTree. The tree was rooted according to the midpoint strategy using python’s scikit-bio package and the resulting rooted tree and corresponding ASV abundances served as an input to scikit-bio PCoA procedure.

### Statistical Analyses

Differences in the Shannon α-diversity measurements, community phenotypes including; SCFA, sugar utilization pathways, vitamin production, glycosyl hydrolase and the relative abundance of particular taxa in our study groups when compared to controls were assessed using a double-tailed Mann–Whitney or *t*-test. Significant differences in the frequencies of taxa present between groups were assessed using a double tailed chi-squared test.

### Genome Reconstruction of SCFA Synthesis, Amino Acid and Sugar Utilization Pathways, and Glycosyl Hydrolases

To predict metabolic capabilities of microbial taxa identified by 16S analysis we used a subsystem-based approach implemented in microbial community SEED (mcSEED), an application of the SEED genomic platform (Overbeek et al., 2014) that have been used to capture, analyze and extend pathways, enzymes, and transporters involved in specific subsystems (metabolic pathways) in the reference set of 2,662 genomes representing 690 microbial species from human gut. The subsystem-based approach implement functional gene annotation and prediction using three comparative genomic techniques: (i) homology-based methods; (ii) genome context analysis; (iii) co-regulation by the same regulon (Wattam et al., 2014).

The collection of curated metabolic subsystems analyzed for this work includes a subset of catabolic pathways for various sugars ( mono-, di-, and oligosaccharides) and amino acids, as well as two SCFA synthesis pathways (propionate and butyrate). The analyzed microbial genomes were imported to mcSEED from the PATRIC genomic database (Ravcheev et al., 2013). The metabolic subsystems were developed based on previously published genomic studies of sugar and amino acid metabolism in various bacterial taxa (Gu et al., 2010; Ravcheev et al., 2013; Rodionova et al., 2013; Arzamasov et al., 2018; Bouvier et al., 2018) and the studies of phylogenetic distribution of bacterial pathways for production of butyrate (Vital et al., 2014) and propionate (Reichardt et al., 2014). As result, each reference genome in each analyzed subsystem was assigned a binary (“1” or “0”) phenotype reflecting the presence/absence of a complete sugar/amino acid catabolic or SCFA synthesis pathway. Binary phenotypes in reference genomes were summarized in the form of a Binary Phenotype Matrix (BPM). In addition to catabolic enzymes, the sugar utilization subsystems also included sugar-specific uptake transporters, thus the assigned sugar utilization capability required the presence of both catabolic pathway and uptake transporter (Centers for Disease Control, 1986). The distribution of carbohydrate active enzymes (CAZymes) including 229 families of glycosyl hydrolases and 57 families of pectate lyases in the analyzed reference genomes was obtained using dbCAN2 tool (Zhang et al., 2018). The obtained CAZyme family distribution was made converted to a binary GH-BPM matrix of appearance of each GH family across the analyzed reference genomes. The obtained BPM and GH-BPM for metabolic phenotypes and CAZyme family distributions for 2,662 reference genomes provided as a part of the Phenotype Profiler tool were used to calculate a community phenotype matrix for all mapped taxa obtained from 16S analysis as previously described (Rodionov et al., 2019). Community phenotype index (CPI) for each 16S sample was further calculated as a sum of respective community phenotype matrix values of each taxa multiplied by their relative abundances. CPI provides a fractional representation of cells in the community possessing a specific metabolic pathway or CAZyme family (on the scale 0–100%). The Phenotype Profiler tool for CPI calculation was provided by PhenoBiome Inc. (San Francisco, CA, United States).

## Results

### Medicinal Herbs Modulate Fecal Microbiota

We used anaerobic fecal cultivation to evaluate the modulatory effects of 11 herbs used in Ayurvedic medicine. To maximize phylogenetic diversity of microbial communities, we analyzed fecal inoculums generated by pooling equal masses of stool collected from 12 healthy vegetarian donors. We compared the composition of fecal communities in a CDM lacking any carbohydrate source (control) to CDM supplemented with 1% medicinal herbs (w/v). CDM provides all 20 amino acids that positively select for taxa capable of fermenting amino acids for energy and cellular growth.

Compared to control cultures (*n* = 3), Herb-supplemented cultures (*n* = 4–6) exert positive selection of taxa capable of amino acid fermentation and those capable of utilizing herb constituents for energy. We profiled resulting communities by sequencing amplicons featuring the V3–V4 region of the 16S rRNA gene followed by analysis of taxa present in each community. In total, we profiled 414 distinct phylotypes among all cultures analyzed (Supplementary Table 1A). We define a phylotype as a unique sequence differing by as little as 1 base, that approximate strain/species level assignments. This analytical approach allowed us to assess strain-level variability in response to each herb and to determine the statistical significance of herb-induced changes (Supplementary Table 1B).

**FIGURE 1:**
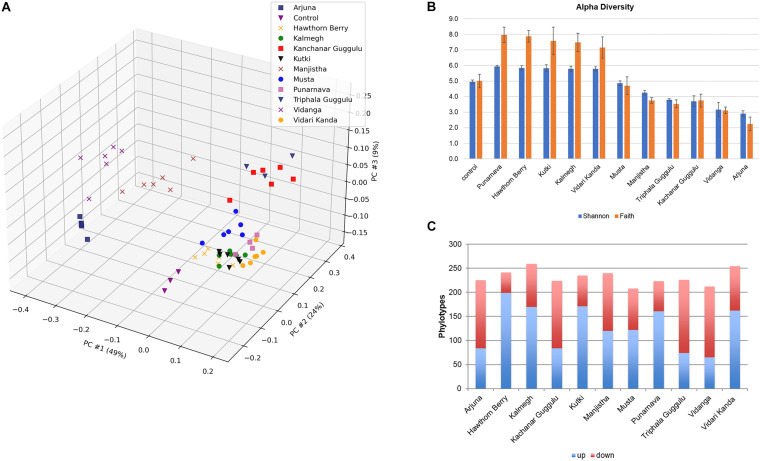
**(A)** Principal Components Analysis (PCoA) based on weighted UniFrac distance, β-diversity. Control cultures compared to cultures supplemented with medicinal herbs. **(B)** Shannon α-diversity of herb-supplemented communities. Data is compared to control cultures and presented in order of decreasing α-diversity. **(C)** Modulatory effects of medicinal herbs. The average relative abundance of phylotypes generated in herb-supplemented cultures were compared to control cultures. Taxa displaying five-fold increased relative abundance (blue) and five-fold reduced relative abundance (red).

To evaluate the modulatory capacity of each medicinal herb, we performed β-diversity measures using weighted UniFrac distances of each culture to assess the overall relatedness of herb-supplemented cultures compared to control cultures as shown by PCoA (Figure 1A). Each herb altered the composition of fecal communities to varying extents relative to control cultures. Herb-selected communities generated in technical replicates resulted in closely related communities, although some clustered more tightly than others. Herb-supplemented cultures formed three groups. Cluster 1 included; Hawthorn Berry, Kalmegh, Kutki, Musta, Punanarva, and Vidari Kanda. Common to these communities were a relatively balanced structure comprised of multiple bacterial families. A second cluster formed by communities supplemented with Triphala Guggulu and Kanchanar Guggulu shared a strong expansion of Bacteroidaceae driven by members of the genus Bacteroides. A third loosely grouped cluster formed by communities supplemented with Arjuna, Manjistha, and Vidanga were driven by strong expansion of Enterobacteriaceae and differential expansion of Enterobacter (Arjuna and Manjistha). The separation of Vidanga-supplemented cultures was driven by an expansion of the genera *Escherichia* and *Acidaminicoccus*.

To assess the impact of medicinal herbs on microbial diversity, we used Shannon diversity index measures and found that cultures supplemented with Punarnava (*p* < 0.0001), Kutki (*p* < 0.0001), Vidari Kanda (*p* < 0.0001), Hawthorn Berry (*p* < 0.0001), and Kalmegh (*p* < 0.0001) resulted in an expansion of α-diversity compared to control cultures (Figure 1B). This was consistent with the observation that these cultures displayed an increase in the number of taxa displaying elevated relative abundance (>5-fold) and a relatively smaller number of taxa displaying reduced relative abundance compared to control cultures (Figure 1C and Supplementary Table 1C).

Many taxa enriched by herbs were not detected in control cultures and frequently associated with very large fold-increases. We note that Musta, Kanchanar Guggulu, Triphala Guggulu, Arjuna, and Vidanga-supplemented cultures displayed a disproportionate number of taxa displaying reduced relative abundance compared to control cultures. Manjistha increased the relative abundance of approximately the same number of taxa as it decreased. Based on this data, it is not possible to establish whether the effect of herbs that predominantly reduced relative abundance of taxa reflect inhibitory properties of those herbs. These results indicate that each medicinal herb tested possessed strong capacity to modulate microbial community composition with overall herb-specific effects.

### Phylogenetic Distribution of Herb-Selected Microbial Communities

**FIGURE 2:**
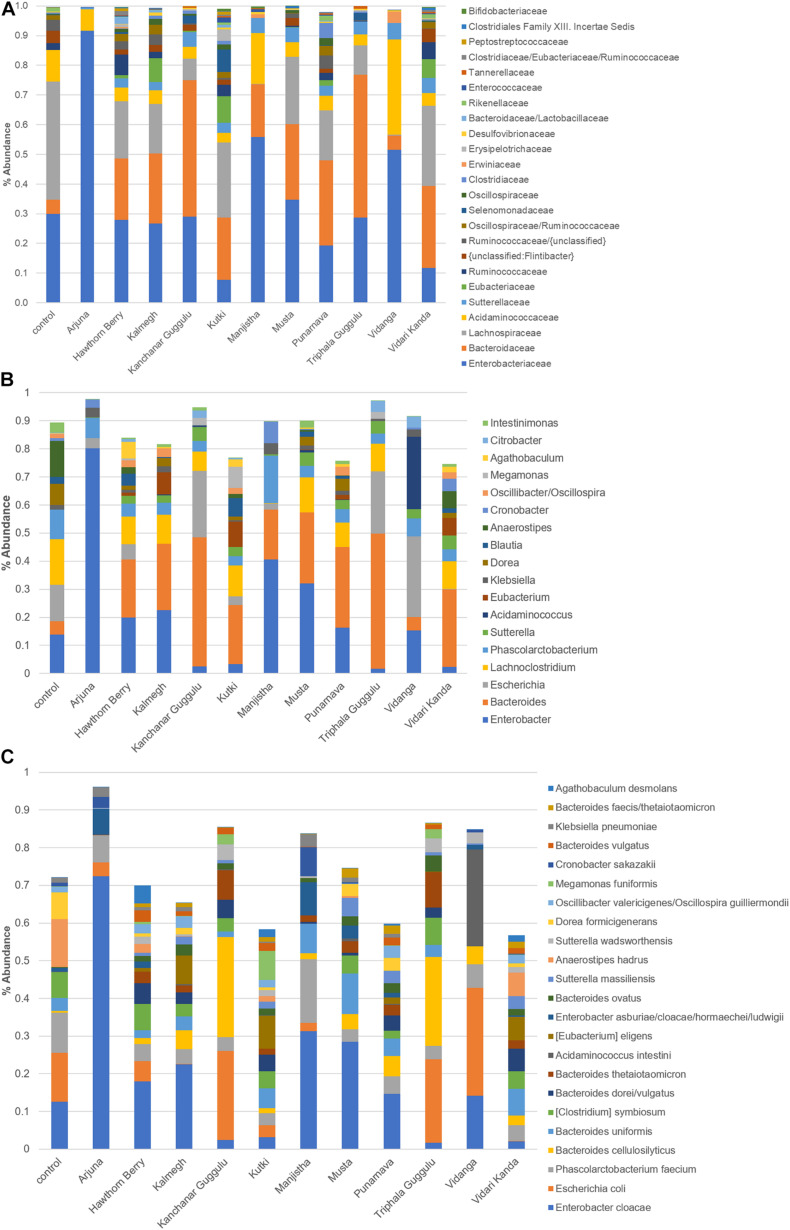
**(A)** Distribution of taxa present in medicinal herb-selected communities. The relative abundance of each family was summed and is presented as % abundance of the total. **(B)** Relative abundance of dominant genera in herb-selected communities. Relative abundance is shown as % of total for each genus. **(C)** Relative abundance of dominant species in herb-selected communities. Relative abundance of the most prevalent species is shown as% of total. Color key is read left to right, corresponding to taxa from bottom to top.

We further analyzed communities generated by each medicinal herb at the family level (Figure 2A). This analysis highlighted that each herb uniquely altered fecal communities as the result of positive selection for microbes with the capacity to metabolize herb components and those benefiting from cross-feeding on metabolites generated by primary herb catabolizers. These alterations indicate those taxa driving observed changes in β-diversity shown in Figure 1A. Several herbs increased the relative abundance of Bacteroidaceae, primarily the result of expansion of the genus *Bacteroides*. These changes were particularly evident in cultures supplemented with Kanchanar Guggulu and Triphala Guggulu and to a lesser extent in cultures supplemented with Hawthorn Berry, Kalmegh, Kutki, Manjistha, Musta, and Vidari Kanda. This effect was not observed in cultures supplemented with Arjuna or Vidanga.

Compared to control cultures that were dominated by Lachnospiraceae (∼40% relative abundance) and organisms that ferment amino acids for energy production, all herb-supplemented cultures displayed reduced relative abundance of this family, although Kutki (25%), Musta (23%), and Vidari Kanda (27%) supplemented cultures, maintained Lachnospiraceae at relatively high levels. By contrast, cultures supplemented with Arjuna, Manjistha, and Vidanga were essentially devoid of Lachnospiraceae. Some of the herbs tested including, Hawthorn Berry (7.1%), Vidari Kanda (5.7%), and Punarnava (2.6%) modestly expanded the representation of Ruminococcaceae compared to control cultures (2.2%), whereas, Arjuna (0.0%), Kanchanar Guggulu (0.03%), Manjistha (0.0%), Musta (0.43%), Vidanga (0.003%), and Triphala Guggulu (0.13%) reduced the representation of this family. Punarnava (5.0%). Kalmegh (1.1%), Kanchanar Guggulu (1.2%), and Kutki (1.3%) increased the relative abundance of Clostridiaceae compared to control cultures (0.33%). Eubacteriaceae was not detected in control cultures but was expanded in cultures supplemented with Hawthorn Berry (1.1%), Kalmegh (7.9%), Kutki (9%), Punarnava (1.8%), and Vidari Kanda (6.2%). Finally, we note that compared to control cultures (30%), Kutki (7.8%), and Vidari Kanda-supplemented cultures (11.7%) reduced the representation of Enterobacteriaceae, whereas Arjuna (91.6%), Manjistha (55.8%), and Vidanga-supplemented cultures (51.5%) were dominated by this family. We note that PCoA and phylogenetic changes induced by Kanchanar Guggulu and Triphala Guggulu were highly similar, suggesting that the Guggulu in these herbal formulations was largely responsible for the changes observed.

### Genus and Species-Level Representation in Herb-Supplemented Communities

Individual phylotypes with high sequence similarity to named species in control and herb-supplemented cultures generated highly coherent induction/repression responses (Supplemental Table 1C). Amino acids represent the sole energy source present in control media. Therefore, the most abundant taxa present in control cultures represent the most efficient amino acid fermenters (Supplementary Table 2). We note that the vast majority of the 71 phylotypes displaying a relative abundance of >0.1% of the total community in control cultures are unaltered or reduced in abundance in herb-supplemented cultures. This result suggests a fundamental shift in community metabolism of herb-supplemented cultures.

The most abundant genera profiled in control cultures was *Anaerostipes* primarily due to the occurrence of *Anaerostipes hadrus* (13%), a butyrate producing species that consumes lactate and acetate. The relative abundance of *A. hadrus* was reduced in all herb-supplemented cultures Hawthorn Berry (2.3%), Kutki (1.4%), and Vidari Kanda (6.1%), and sharply reduced in the remaining herb cultures (range = 0.5–0.001%) (Figures 2B,C). *Enterobacter* primarily due to *Enterobacter cloacae*, was prevalent in control cultures (13%) and was further increased by Arjuna (72%), Manjistha (31%), Musta (29%), and Kalmegh (23%), whereas Kanchanar Guggulu (2.5%), Kutki (3.2%), Triphala Guggulu (1.7%), and Vidari Kanda-supplemented cultures (2.0%) reduced the relative abundance of this species (Supplementary Table 1C). Compared to control cultures (0.01%), the relative abundance of phylotypes related to *Eubacterium eligens* were uniformly increased in cultures supplemented with Hawthorn Berry (0.9%), Kalmegh (7.7%), Kutki (8.7%), Punarnava (1.7%), and Vidari Kanda 6.2%). *E. eligens* is known to metabolize complex carbohydrates and is a butyrate producer and associated with gut health (Pryde et al., 2002). The *Bacteroides* are only modestly represented in control cultures (4.9%) but undergo a strong expansion in most herb-supplemented cultures, particularly in those supplemented with Kanchanar Guggulu (46%), Punarnava (28.7%), and Triphala Guggulu (48.1%) and to a lesser extent in cultures supplemented with Hawthorn Berry (20.7%), Kalmegh (23.6%), Kutki (20.9%), Manjistha (17.8%), Musta (25.4%), and Vidari Kanda (27.6%).

*Bacteroides* were observed at the limit of detection in cultures supplemented with Arjuna. Many *Bacteroides* reside in the mucin layer where they forage on host glycans (Tailford et al., 2015) and have evolved to have an intimate interaction with the immune system. Among the notable activities of species belonging to this genus include; increased gut epithelium barrier function, induction of tolerance and anti-inflammatory cytokines (Round and Mazmanian, 2010; Hsiao et al., 2013; Erturk-Hasdemir et al., 2019). LPS is a potent pro-inflammatory ligand, however the pentacylated form of LPS encoded by *Bacteroides* spp. are weak agonists of TLR4 signaling and provide a protective effect restoring gut immune homeostasis (Steimle et al., 2019). Among the 19 *Bacteroides* spp., observed, most were strongly increased by herb-supplementation, including; *Bacteroides cellulosilyticus*, *Bacteroides faecis*, *Bacteroides thetaiotaomicron*, *Bacteroides vulgatus*, and *Bacteroides xylanisolvens*. The relative abundance of most phylotypes related to *Bacteroides intestinalis* and *Bacteroides uniformis* was unchanged. Phylotypes with high similarity to *Parabacteroides* spp. were increased in several herb-supplemented cultures (0.2–1%) compared to control cultures (0.001%) with the exception of Arjuna, Hawthorn Berry, Musta, and Vidanga.

*Blautia* spp. consume H_2_ generated from fermentation of sugars, relieving the inhibition of further fermentation reactions and displayed increased relative abundance in an herb-specific manner. This genus has been associated with improved barrier function and reduced inflammation in models of murine ulcerative colitis in response to chitosan supplementation (Wang et al., 2019). *Blautia producta* reduced poly (I:C)-induced inflammatory cytokine expression in HT-29 cells (Pathmanathan et al., 2020). Hawthorn Berry (4.3%) and Kutki (6.7%) broadly increased most *Blautia* phylotypes compared to control cultures (2.5%), whereas Musta and Vidari Kanda displayed phylotypes displaying both increased and decreased relative abundance relative to control cultures. The remaining herbs had no effect or displayed decreased relative abundance of *Blautia* spp. Another group of H_2_ consumers are *Desulfovibrio* spp. namely, *Desulfovibrio desulfuricans* and *Desulfovibrio piger*. *Desulfovibrio* displayed reduced abundance in constipated-predominant IBS patients (Gobert et al., 2016). These phylotypes were detected in control cultures (0.35%) and were increased in cultures supplemented with Kalmegh (1%). All other herbs tested displayed an increase in *D. desulfuricans* and a decrease in *D. piger*.

*Dorea* spp. were reduced in relative abundance by all herbs (0.0–4.3%) compared to control cultures (7.5%). *Dorea* spp. was reduced in abundance following whole strawberry feeding in a DSS-induced colonic inflammatory colitis (Han et al., 2019). Numerous phylotypes with high similarity to *Oscillibacter* spp. were differentially altered. A study investigating the impact of DPP-4 that increases the half-life of incretins and restores intestinal homeostasis were associated with decreased abundance of *Oscillibacter* (Olivares et al., 2018). Compared to control cultures (1.5%), the relative abundance of *Oscillibacter valericigenes* was increased by Hawthorn Berry (2.5%), Kalmegh (3.1%), Kutki (2%), Punarnava (3.2%), and Vidari Kanda (2.3%), whereas Arjuna, Triphala Guggulu, and Vidanga (0.01%) primarily decreased phylotypes related to this species.

Based on analysis of sugar utilization potential of *O. valericigenes*, we find that this species is uniquely capable of utilizing arabinose and xylose. Phylotypes related to two *Phascolarctobacterium* spp., *Phascolarctobacterium faecium* and *Phascolarctobacterium succinatutens* also are predicted to encode limited sugar utilization capacity (fructose and ribose) are largely unaltered by herb-supplementation with some sporadic cases of reduced relative abundance among some phylotypes. The relative abundance of phylotypes with similarity to *Parasutterella* and *Sutterella* were uniformly elevated by all herbs tested. This result is unexpected given that species belonging to these genera can only utilize fructose, a sugar not commonly found in medicinal herbs (Peterson et al., 2019a,b).

We analyzed phylotypes belonging to Enterobacteriaceae since many but not all species in this family are considered pathobionts and may contribute to elevated inflammation in the host. We have previously noted that the relative abundance of taxa belonging to this family often reach relative abundance levels higher than that commonly observed *in vivo*. Phylotypes related to *Citrobacter* spp., were in low abundance in control cultures (0.2%) and were largely unaffected by herb-supplementation with the exception of Kanchanar Guggulu (2.7%), Triphala Guggulu (4.0%), and Vidanga (4.0%) that increased the relative abundance of most phylotypes. Arjuna (3.1%), Manjistha (7.7%), Vidari Kanda (4.4%) increased the relative abundance of *Cronobacter* spp. compared to control cultures (1%). Other herb-supplemented cultures resulted in reduced relative abundance of these species (0–0.7%). The *Enterobacter* were relatively prevalent in control cultures (14%). These phylotypes were reduced in relative abundance by cultures supplemented with Kanchanar Guggulu (2.5%), Kutki (3.4%), Triphala Guggulu (1.7%), and Vidari Kanda (2.4%) but strongly increased by Arjuna (80%). Among the other medicinal herbs tested, changes in abundance were not observed.

Compared to control cultures (13%), Arjuna (3.7%), Hawthorn Berry (5.4%) Kalmegh (0.04%), Kutki (3.2%), Musta (0.001%), Punarnava (0.001%), and Vidari Kanda-supplemented cultures (0.1%) displayed a reduced relative abundance of *Escherichia* whereas, Kanchanar Guggulu (23.6%), Triphala Guggulu (22.1%), and Vidanga (28.7%) increased the relative abundance of this genus. Phylotypes closely related to *Klebsiella* spp. were observed in control cultures (1.6%) and were reduced in relative abundance in cultures supplemented with Kanchanar Guggulu (0.15%), Triphala Guggulu (0.74%), and Vidari Kanda (0.04%), whereas other herbs generally did not alter the relative abundance of this group of pathobionts with the exception of Manjistha (4%).

### Short Chain Fatty Acid Production Capacity of Herb-Induced Communities

To derive functional information based on 16S rRNA profiled communities, we used genome reconstruction of over 2,200 reference genomes relevant to human microbiomes to assess butyrate and propionate production capacity in herb-selected communities. The biosynthesis of both butyrate and propionate occurs via four known pathways for each. In the case of butyrate, two pathways involve central carbohydrate metabolism intermediates, whereas the remaining two pathways convert lysine or glutamine to butyrate. Therefore, we considered all potential butyrate biosynthetic pathways relevant for herb-supplemented cultures, whereas only amino acid conversion pathways are relevant to control cultures.

**FIGURE 3:**
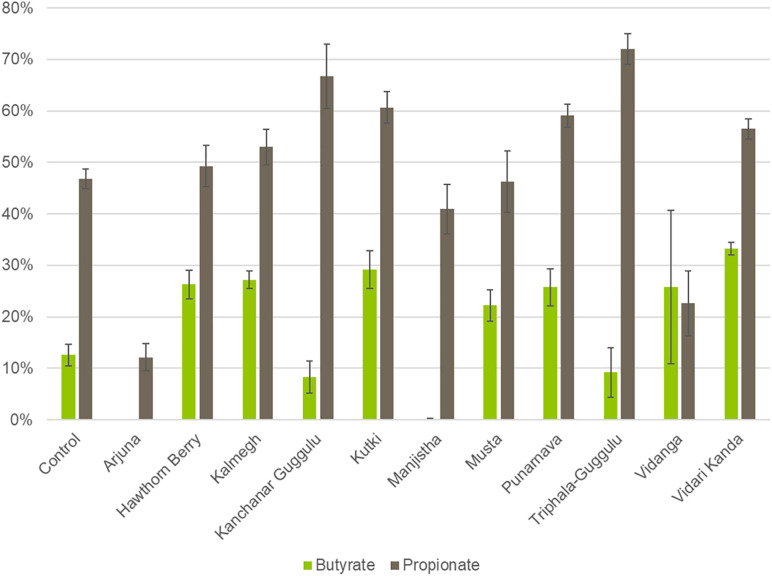
Predicted Butyrate and Propionate production capacity of herb-selected communities. Binary phenotype predictions for observed taxa with identity to >2,200 HMP reference genomes and multiplied by the relative abundance of each. Butyrate and propionate community production capacity is depicted as the sum of the relative abundance-phenotype predictions.

We were able to predict butyrate and propionate biosynthetic potential for the majority of taxa observed at the species level. We summed the relative abundance of SCFA producers to generate predictions of individual communities biosynthetic capacity. Cultures supplemented with Hawthorn Berry (*p* = 0.0007), Kalmegh (*p* < 0.0001), Kutki (*p* = 0.0001), Musta (*p* = 0.0005), Punanarva (*p* = 0.007), and Vidari Kanda (*p* < 0.0001) increase the representation of butyrate producers compared to control cultures, whereas Arjuna (*p* = 0.0007), Kanchanar Guggulu (*p* = 0.02), and Manjistha (*p* < 0.0001) displayed statistically significant reduction in the relative abundance of butyrate producing taxa (Figure 3). It should be noted that the average butyrate biosynthesis capacity predicted for 313 HMP stool samples is ∼25% (unpublished data). Compared to control cultures, several herb supplemented cultures resulted in an increase in the relative abundance of propionate producing taxa, including; Kalmegh (*p* = 0.01), Kanchanar Guggulu (*p* = 0.0004), Kutki (*p* = 0.0001), Punanarva (*p* = 0.0008), Triphala Guggulu (*p* = 0.001), and Vidari Kanda (*p* = 0.001).

### Vitamin Biosynthesis Potential of Herb-Supplemented Cultures

The gut microbiota is known to harbor a relatively large proportion of B vitamin auxotrophs (Rodionov et al., 2019; Sharma et al., 2019). This finding is surprising since B vitamin biosynthetic pathways are considered essential for all life forms since B vitamins are intermediates in the biosynthesis of essential co-factors such as: TTP (B1), FMN/FAD (B2), NAD(P) (B3), etc. We previously demonstrated that B vitamin prototrophs share B vitamins with their auxotrophic neighbors providing stability to gut microbial communities in the face of varied B vitamin provisions in the diet (Sharma et al., 2019).

**FIGURE 4:**
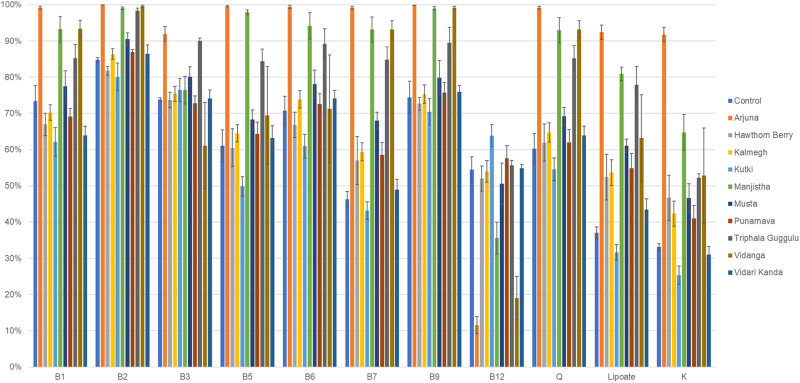
Predicted vitamin biosynthetic potential. Binary phenotype predictions for observed taxa with identity to >2,200 HMP reference genomes and multiplied by the relative abundance of each. Vitamin community production capacity is depicted as the sum of the relative abundance-phenotype predictions.

We therefore evaluated the effect of herb-supplementation on the proportion of B vitamin prototrophs in microbial communities in control and herb-supplemented cultures. To provide an external frame of reference, we included the average prototroph abundances predicted from 313 fecal samples derived from the human microbiome project (HMP). Control cultures were in strong agreement with HMP data for vitamins B1, B2, B12, and Q, whereas control cultures were reduced for the remaining vitamins compared to human data. Compared to control cultures, the herb-supplemented cultures, Arjuna strongly increased all vitamin production capacities (*p* = 0.01– <0.0001), with the exception of vitamin B12 (*p* = 0.001), which was sharply reduced (Figure 4). Hawthorn Berry, Kalmegh, Musta-supplemented cultures modestly increased the predicted prototroph abundance of vitamins B7 (*p* = 0.01, 0.002, and <0.0001, respectively), lipoic acid (*p* < 0.0001, *p* = 0.002, and *p* < 0.0001, respectively), and vitamin K (*p* = 0.004, *p* = 0.001, and *p* < 0.0001, respectively). Kanchanar Guggulu supplementation increased all vitamins to varying extents, except B12 most significantly impacting B5 (*p* = 0.01), B7 (*p* < 0.0001), Q (*p* = 0.008), and lipoate (*p* < 0.0001). Manjistha-supplemented cultures were predicted to increase all vitamins (*p* = 0.02– <0.0001) except B12. Levels of B7 did not achieve statistical significance. Triphala Guggulu is predicted to increase B5 (*p* = 0.005), B7 (*p* = 0.001), Q (*p* = 0.003), and lipoate (*p* = 0.004), whereas Vidanga increased B7 (*p* < 0.0001), B9 (*p* = 0.02), Q (*p* = 0.004), and Lipoate (*p* = 0.004) but reduced the predicted relative abundance of B12 prototrophs (*p* < 0.0001). Punanarva, Kutki, and Vidari Kanda did not alter the average abundance of predicted vitamin prototrophy abundance substantially, although some changes were statistically significant (Supplementary Table 1).

### Amino Acid Production Capacity

**FIGURE 5:**
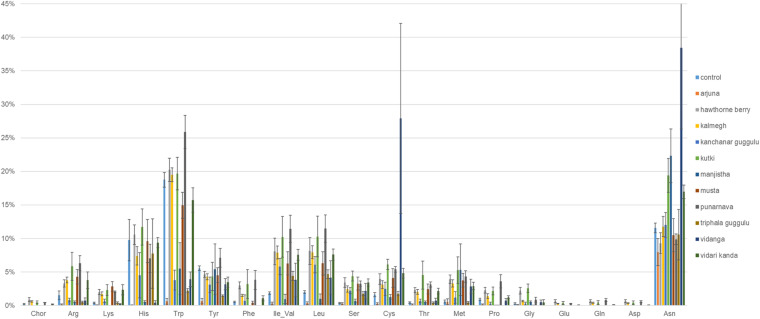
Predicted amino acid biosynthesis potential. The sum of the relative abundance of taxa auxotrophic for amino acid biosynthesis multiplied by predicted binary phenotypes.

We performed genome reconstruction of amino acid biosynthetic pathways to determine the extent to which herb-supplementation alters the representation of amino acid auxotrophs in fecal communities (Figure 5). It is notable that control cultures harbored communities with an average relative abundance of 10 and 19% auxotrophy for Histidine and Tryptophan biosynthesis, respectively. Cultures supplemented with Arjuna, Manjistha, or Vidanga (all *p* = 0.05) decreased the proportion of Histidine auxotrophs, whereas Arjuna (*p* = 0.01), Kanchanar Guggulu (*p* < 0.0001), Manjistha (*p* < 0.0001), Triphala Guggulu (*p* = 0.001), and Vidari Kanda-supplemented cultures (*p* = 0.04) decreased the relative abundance of Tryptophan auxotrophs. Conversely, cultures supplemented with Punarnava (*p* = 0.009; *p* < 0.0001) and Vidanga (*p* < 0.0001; *p* = 0.009) elevated the frequency of Tryptophan and Cysteine auxotrophs, respectively. Vidanga (*p* = 0.004), Manjistha (*p* = 0.001), and Kutki (*p* = 0.001) supplemented cultures all increased the average relative abundance of Asparagine auxotrophs compared to control cultures.

### Glycosyl Hydrolase Representation

All of the medicinal herbs tested had a substantial effect on the representation of glycosyl hydrolase loci abundance. The medicinal herbs tested here overwhelmingly increased the relative abundance of GH loci, although some herbs reduced the abundance of specific GH families compared to control cultures. The net effect (average of positive and negative changes) for all herb-supplemented cultures was positive. Vidanga has the smallest net effect, increasing GH loci abundance by 1%, whereas Arjuna (20%), Kanchanar Guggulu (21%), and Triphala Guggulu (22%) had the greatest effect on the representation of glycosyl hydrolase loci abundance (Supplementary Tables 1, 3).

**FIGURE 6:**
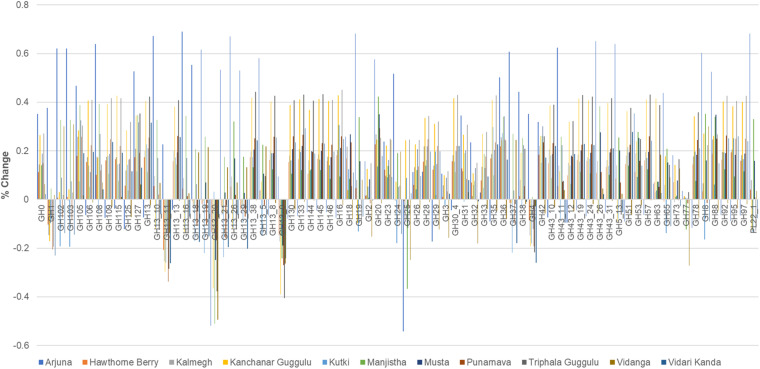
Glycosyl hydrolase family representation. The abundance of abundant glycosyl hydrolase (GH) families (>0.2 total average abundance) are shown relative to the abundance of each GH family relative to control cultures as increased or decreased.

Arjuna stands out as having the largest number of incidences of large positive magnitude increase (>40%), Kanchanar Guggulu and Triphala Guggulu displayed the highest number of cases of increased GH family abundance >20% (Figure 6). Hawthorn Berry, Kutki and Vidanga infrequently increased GH family abundance by >20%. While no medicinal herb had uniform effects on GH representation, some GH families were decreased by most medicinal herbs, including GH1, GH4, GH112, GH13 subfamily 9, 11, 20 and GH25. The GH families significantly increased by herb supplementation is too complex to summarize fully, however it is evident that the majority of GH loci were increased in representation in herb-supplemented communities. Some GH families were increased by the majority of herbs tested including; GH13, GH13 subfamily 38, GH16, GH20, GH36, GH42, GH88, GH105, GH127, and GH133.

### Sugar Utilization

**FIGURE 7:**
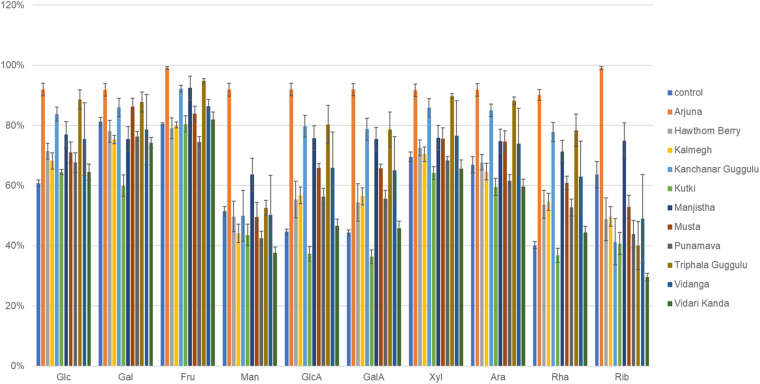
Sugar utilization capacity of herb-selected communities. Binary phenotype predictions for observed taxa with identity to >2,200 HMP reference genomes and multiplied by the relative abundance of each. Sugar utilization capacity is depicted as the sum of the relative abundance-phenotype predictions. Glc, glucose; Gal, galactose; Fru, fructose; Man, mannose; GlcA, glucuronic acid; GalA, galacturonic acid; Xyl, xylose; Ara, arabinose; Rha, rhamnose; Rib, ribose.

We used genome reconstruction to predict sugar utilization capacity of herb-supplemented cultures focused on 10 sugars previously measured by MS in medicinal herbs (Peterson et al., 2019a,b). Among all 14 herbal medicines and culinary spices previously subjected to experimental sugar abundance measurements, each shared in common detectable amounts of each sugar with the exception of mannose which was absent in some medicinal herbs. The herb-selected increased abundance of sugar utilization was highly consistent with characteristics of GH loci selection. Arjuna substantially increased the representation of all sugar utilization pathways (*p* ≤ 0.0001). All sugar utilization pathways increased except for mannose utilization in the case of Kanchanar Guggulu (*p* = 0.03– <0.0001) and galactose and mannose for Triphala Guggulu (*p* = 0.01– <0.0001) as shown in Figure 7. Musta-supplementation mirrored Kanchanar Guggulu, albeit to a lesser magnitude. Manjistha-supplemented cultures increased the abundance of all sugar utilization pathways with the exception of galactose. Punanarva-supplemented cultures modestly increased the representation of glucosamine, galactosamine, and rhamnose utilization pathways. By contrast, Vidari Kanda-supplemented cultures did not have significant positive selection on any sugar utilization pathways and significantly reduced the representation of mannose utilization pathways, despite increasing 24 GH loci by >20%. Similarly, Hawthorn Berry increased the utilization genes of only Rhamnose. This is also consistent with reduced instances of positive selection for GH loci >20% in two instances (Supplementary Table 3). Kutki-supplemented cultures did not alter sugar utilization capacity, consistent with its positive selection of only nine GH families >20%.

## Discussion

We analyzed the modulatory impact of 11 medicinal herbs on fecal bacterial communities *in vitro*. One advantage of using *in vitro* cultivation is the lack of complicating factors such as diet and host dependent effects e.g., the immune system, that may alter the purely microbiological effects of medicinal herbs studied in culture. Here, we made extensive use of genome reconstruction to predict functional potential (phenotypes) of herb-selected communities for a number of relevant biochemical pathways and enzyme systems. Each of the medicinal herbs examined in this study generated substantial changes to the composition of fecal communities. Most herb-selected communities clustered relatively tightly based on β-diversity measurements, although some herbs such as Arjuna, Kalmegh, Kutki, and Musta formed two clusters and Vidari Kanda failed to form discrete clusters. We have observed this phenomenon previously (Peterson et al., 2018, 2019a,b) and suggest that this is the result of stochastic events that favor more than one high fitness community to be selected by these herbs. We speculate that early events in herb catabolism, may favor two or more similarly fit taxa to gain initial dominance thereby dictating differential selection to form distinct communities. This perspective highlights the idea that the metabolism of gut microbes is highly inter-dependent, via secreted metabolites and secondary metabolism networks. More detailed analyses including metabolomic analyses may shed light on the metabolites that drive herb-mediated changes in community structure.

Based on ecological principles, higher α-diversity of microbial communities is considered a healthier state that is more resistant to perturbation, or more resilient. Some of the medicinal herbs we examined increased the α-diversity of resulting communities compared to control cultures, including; Punarnava, Kutki, Vidari Kanda, Hawthorn Berry, and Kalmegh. These results may be explained by the fact that many of the taxa displaying increased relative abundance in herb-supplemented cultures were not detected in control cultures. These taxa could be considered as the most reliable signatures of herb-responsive taxa.

Based on comparisons to control cultures the medicinal herbs analyzed may be divided into three groups. Consistent with α-diversity measures, Hawthorn Berry, Kalmegh, Kutki, Punarnava, and Vidari Kanda were strongly stimulatory, resulting in the increased relative abundance of >150 phylotypes (>5-fold). Conversely, Arjuna, Kanchanar Guggulu, Triphala Guggulu, and Vidanga reduced the relative abundance of a larger number of phylotypes than it increased (range = 140–152). It is tempting to speculate that these herbs possess anti-microbial activities, however examination of these communities reveals that each was associated with 2 or more dominant species (Arjuna: *E. cloacae* 72%; Kanchanar Guggulu: *Escherichia coli* 24%, *Bacteroides cellulolyticus* 27%; Triphala Guggulu: *E. coli* 22%, *B. cellulolyticus* 24%; and Vidanga: *E. coli* 29%, *Acidaminococcus intestini* 26%). The dominant taxa necessarily crowd out a larger number of species. By contrast, herbs with high positive selection (number of phylotypes increased) did not harbor dominant species but rather large arrays of lower abundance species (Figure 2C).

Analysis of herb-supplemented cultures at various phylogenetic levels allows distinctions to be inferred regarding community metabolism (family) and the phenotypic properties of individual species mediating the metabolism of herb substrates. With the exception of Arjuna and Vidanga, all other herb-selected communities featured an expansion of Bacteroidaceae, predominantly *Bacteroides*. Members of *Bacteroides* are metabolically adaptable, capable of amino acid and diverse sugar metabolism. Indeed, large suites of loci encoding glycosyl hydrolases with broad specificity enable these species to catabolize complex carbohydrates that are likely to be prevalent in medicinal herbs.

Conversely, we have noted that cultures supplemented with simple sugars tend to strongly enrich species belonging to Enterobacteriaceae that encode diverse sugar utilization pathways. This family was expanded in cultures supplemented with Arjuna, Manjistha, and Vidanga. We speculate that these herbs may possess elevated levels of simple sugars that sustain high relative abundance of Enterobacteriaceae. Lachnospiraceae represent the dominant family in control cultures. With the exception of Arjuna, Manjistha, and Vidanga where this family was eliminated, other herbs maintained varying quantities of Lachnospiraceae, particularly cultures supplemented with Kutki and Vidari Kanda. The reason(s) for this herb-dependent reduction is unclear, however most of the six species (62% of the community) are reduced in relative abundance in herb-supplemented communities. This suggests that herb-selected communities undergo a shift from amino acid fermentation to herb-substrate metabolism.

The relative abundance of the family Acidaminococcaceae was high in control cultures due to the presence of phylotypes related to *A. intestini*. This microbe generates butyrate through amino acid fermentation. This species encodes sugar utilization pathways for fructose only. While *A. intestini* relative abundance was decreased in most herb-supplemented cultures it was increased in cultures supplemented with Manjistha and Vidanga. This result suggests that the fitness of *A. intestini* in these herb-supplemented cultures may be due to combined amino acid and sugar fermentation.

### Predicted Short Chain Fatty Acid Biosynthesis Potential

The application of genome reconstruction of pathways encoded in microbial genomes for the biosynthesis of butyrate and propionate allowed us to predict the impact of medicinal herbs on these important signaling molecules. Butyrate biosynthesis is carried out by one of four pathways, two involving amino acid fermentation using lysine or glutamate as substrate and two that reflect sugar fermentation that use succinate or pyruvate as substrate. Only the amino acid fermentation pathways are relevant to control cultures since the growth media is devoid of sugar substrates, whereas taxa encoding any of the four butyrate biosynthetic pathways were considered relevant for herb-supplemented cultures.

We noted that several medicinal herbs including Hawthorn Berry, Kalmegh, Kutki, Punanarva, Vidanga, and Vidari Kanda all enriched butyrate producers compared to control cultures. Butyrate is the most well studied metabolite produced by gut microbiota and serves as an important energy source for intestinal epithelial cells and the maintenance of an anaerobic environment in the lumen (Li et al., 2019) and importantly increases gut barrier integrity (Bach Knudsen et al., 2018). Additionally, butyrate is an HDAC inhibitor that leads to an expansion of anti-inflammatory Treg cells. Medicinal herbs capable of increasing butyrate production may therefore be expected to reduce gut and systemic inflammation. By contrast Arjuna, Kanchanar Guggulu, Manjistha, and Triphala Guggulu displayed reduced relative abundance of butyrate producing taxa (Figure 3).

While most of the butyrate produced by gut microbiota is consumed by the gut epithelium, resulting in low concentrations in circulation, propionate is absorbed in the gut and enters circulation at higher concentrations (Hosseini et al., 2011). Propionate has been shown to inhibit cholesterol and fatty acid synthesis by the liver and increase adipogenesis and decrease lipolysis of adipose tissue. Compared to control cultures, several herb supplemented cultures resulted in an increase in the relative abundance of propionate producing taxa, including; Kalmegh, Kanchanar Guggulu, Kutki, Punanarva, Triphala Guggulu, and Vidari Kanda.

### Vitamin Biosynthesis Potential of Herb-Supplemented Cultures

We previously reported the genome reconstruction of 8 B vitamin biosynthetic pathways (Rodionov et al., 2019; Sharma et al., 2019) to demonstrate that GF mice colonized with human microbiota and provided diets lacking all B vitamins, maintained B vitamin auxotrophs with unaltered relative abundance. These results indicate that B vitamin prototrophic organisms provide essential B vitamins to auxotrophic microbes thereby allowing maintenance of stable microbial communities in the face of varied B vitamin dietary intake. The sharing of B vitamins by prototrophs may also represent a potentially important source of B vitamins for the host (Sharma et al., 2019).

We analyzed control and herb-supplemented cultures to establish the predicted relative frequency of B vitamin prototrophs together with additional vitamins K, Q, and α-lipoic acid. With the exception of vitamin B12, several herb-supplemented cultures increased the relative proportion of B vitamin prototrophs, including vitamin K, Q, and α-lipoic acid. We noted that Arjuna strongly increased all vitamin production capacity, with the exception of vitamin B12, which was sharply reduced (Figure 4). This result is largely explained by the very high abundance of the multi-vitamin prototrophy encoded by *E. cloacae*. Hawthorn Berry, Kalmegh, Musta, and Punanarva-supplemented cultures modestly increased the predicted prototroph abundance of vitamins B7, α-lipoic acid, and vitamin K. Kanchanar Guggulu, Manjistha, Triphala Guggulu, and Vidanga positively selected for vitamin prototrophs similar to those observed in Arjuna-supplemented cultures, albeit to a smaller degree. Kutki and Vidari Kanda-supplemented cultures had little effect on any predicted vitamin production capacity despite significant restructuring of fecal communities.

### Amino Acid Production Capacity

Most fecal microbes are prototrophic for the biosynthesis of all amino acids. Therefore, it is not surprising that medicinal herbs did not generally have a substantial effect on these pathways (Figure 5). Since amino acids are present in all cultures, the microbial dependence of amino acid uptake from herb-substrates is confounded. Insights pertaining to the role of amino acid uptake from herbs and their potential modulatory effect on fecal community composition is a subject of interest and will require the analysis of cultures in media lacking one, several and all amino acids.

### Herb Polysaccharides and Sugars Drive Prebiotic Effects

Virtually all bacteria present in gut microbiota encode glycosyl hydrolases, although some microbes have dramatically expanded these functions to equip them with the capacity to efficiently break down complex polysaccharides available in the host diet. These functions are thought to represent a key feature of gut microbiota illustrating mutualism with their hosts as the human genome encodes relatively few such enzymes. Therefore, the human host depends on the gut microbiota for these functions and it has been estimated that as much as 10% of the calories derived from diet are the result of GH activity that liberate simple sugars from complex dietary fibers that simultaneously cross-feed other microbes that may lack relevant GH functions. The specificities of GH families are imprecise and often encompass multiple substrates, however the overall increase in GH family representation in herb-supplemented cultures support the conclusion that polysaccharides present in herbs may be a key driver of the modulatory effects observed for each medicinal herb examined.

We used genome reconstruction to predict sugar utilization capacity of herb-supplemented cultures. Arjuna-supplemented cultures displayed a significant reduction in the relative abundance of numerous taxa, reflected as low diversity communities (Figures 1B,C), yet this herb increased the sugar utilization potential for all sugars (Figure 7). This result may be explained by the significant bloom of *E. cloacae* that encodes numerous sugar utilization pathways. Triphala Guggulu also increased sugar utilization pathways for all sugars. By contrast to Arjuna, the observed increases in sugar utilization potential is more distributed involving multiple induced taxa. With the exception of mannose, Kanchanar Guggulu increased the representation of all sugar utilization pathways. This result is also distributed across multiple taxa but to a large degree may be explained by the increased relative abundance of *E. coli* and *B. cellulolyticus*, both of which encode broad sugar utilization capacity.

Hawthorn Berry and Kalmegh did not alter sugar utilization gene abundance significantly except for a small increase rhamnose related genes for both, whereas Kalmegh also increased glucosamine utilization. Manjistha-supplemented cultures increased the abundance of glucose, mannose, glucosamine, galactosamine, and rhamnose utilization pathways. Musta and Punanarva-supplemented cultures increased the representation of glucosamine, galactosamine, and rhamnose utilization pathways. It should be emphasized that the lack of change in sugar utilization in Vidari Kanda-supplemented cultures does not necessarily indicate that the modulatory effects of this herb are not based on sugar fermentation since many of the dominant bacteria present in control cultures are dualists, capable of deriving energy from both amino acid and sugar fermentation.

The work described here illustrates that medicinal herbs used to promote immune homeostasis possess inherent prebiotic qualities as evidenced by the strong modulatory capacity that restructure fecal communities and their community metabolism of medicinal herb substrates. Each medicinal herb selected for unique bacterial communities, although some medicinal herbs displayed strong relationships with respect to the communities they selected, e.g., Manjistha and Arjuna. Several medicinal herbs including; Punarnava, Kutki, Vidari Kanda, Hawthorn Berry, and Kalmegh served to increase the α-diversity of communities due significantly to the positive selection of numerous taxa not observed in control cultures. Other medicinal herbs reduced α-diversity including; Manjistha, Triphala Guggulu, Kanchanar Guggulu, Vidanga, and Arjuna. This was due to the strong positive selection of one or a few dominant taxa.

We applied genome reconstruction to enable predictions of metabolic capacity of medicinal herb-selected microbial communities and determined that medicinal herbs altered the representation of pathways involved in butyrate and propionate biosynthetic potential, particularly of the latter. Medicinal herbs selected for communities that overall increased the proportion of taxa capable of synthesizing B vitamins, Vitamins K and Q and α-lipoic acid. The impact of medicinal herbs on amino acid biosynthetic potential was less evident owing to the high proportion of amino acid prototrophs in all cultures analyzed. The most profound effects of medicinal herbs related to the restructuring and increased representation of a large number of glycosyl hydrolase loci that liberate simple sugars from complex carbohydrates present in medicinal herbs. This was consistent with the positive selection of pathways involved in sugar utilization. Taken together, our results suggest that polysaccharides and their associated sugar components represent a significant driving force responsible for the observed restructuring of fecal bacterial communities.

While we acknowledge the limitations of *in vitro* assessments of medicinal herbs, the facile nature of this approach provides significant insights to community metabolism induced by medicinal herb substrates and provides a rational basis for selecting those medicinal herbs of interest for testing *in vivo*. We elected to use fecal pools for our assessments to maximize the diversity of taxa analyzed with the acknowledged trade-off that we did not evaluate inter-personal variability in herb-responses that we consider the subject of future studies. Another potential limitation in interpreting *in vitro* data is that herbs may contain substantial quantities of simple sugars that would otherwise be absorbed in the small intestine, thereby minimizing their influence on colonic or fecal communities.

Mounting evidence strongly support the intimate interactions of the gut microbiome and host immune system. The medicinal herbs studied here have been used for a wide variety of health applications but are generally unified by their impact on the immune system. Our results provide evidence in support of the hypothesis that the therapeutic efficacy of these herbs may be manifested through the intermediary of the gut microbiome. Future *in vivo* studies are required to confirm this hypothesis and to determine the extent that microbiota in herb-supplemented human interventions differ compared to observations made *in vitro*.

## Data Availability Statement

The datasets presented in this study can be found in online repositories. The names of the repository/repositories and accession number(s) can be found below: https://www.ncbi.nlm.nih.gov/genbank/. The Bioproject ID is PRJNA633107 and accession numbers are SAMN14933034–SAMN14933091.

## Ethics Statement

The studies involving human participants were reviewed and approved by Sanford Burnham Prebys Medical Discovery Institute Institutional Review Board (IRB-2014-020). The patients/participants provided their written informed consent to participate in this study.

## Author Contributions

CP conceived the study, contributed to the data generation, analysis, and interpretation, wrote the manuscript, and provided the funding. SU contributed to the data generation. DR and SI provided the bioinformatics tools, contributed to the data analysis, and wrote the manuscript. SP contributed to the data analysis, data interpretation, and writing of the manuscript. JP-S performed the statistical analyses. All authors were involved with biological interpretation, reviewed the manuscript, revised the manuscript, and approved of the final version for publication.

## Conflict of Interest

DC is a founder of the Chopra Foundation and Chopra Center and a co-owner of the Chopra Center. DR is a co-founder of PhenoBiome Inc. The remaining authors declare that the research was conducted in the absence of any commercial or financial relationships that could be construed as a potential conflict of interest.
