# Supplementary material for: Community Metabolic Interactions, Vitamin Production and Prebiotic Potential of Medicinal Herbs Used for Immunomodulation
Source: Front Genet. 2021 Feb 3;12:584197. doi: 10.3389/fgene.2021.584197 (PMC7886795; doi:10.3389/fgene.2021.584197)
Supplement: Supplementary file 1 [file Table_1.DOCX]

Supplementary Material

**Supplementary Table Legends**

**Table S1A. 16S rRNA profiles of control and herb-supplemented communities.** Sequence reads were normalized for each sample by converting read counts into % relative abundance. Relative abundance of each sample is shown together with the average relative abundance. Individual phylotypes and % relative abundance observed in control and herb-selected microbial communities in replicate cultures.

**Table S1B. Statistical analysis of differences of herb-selected microbial communities.** Herb-supplemented cultures were compared to control cultures. Results for each herb are presented in separate tabs and include from top to bottom: GH hydrolase loci abundance, sugar utilization, Shannon diversity, SCFA biosynthetic potential and bacterial phylotypes profiled. Statistical analysis of herb-supplemented culture technical replicates.

**Table S1C. Alterations in fecal microbiota *in vitro.*** Each phylotype and the average relative abundance of control cultures is compared to the average relative abundance of herb-supplemented cultures. Taxa increased >5-fold relative to controls are highlighted in green, taxa decreased >5-fold relative to controls are highlighted in red, taxa changed by <5-fold in yellow and taxa not observed in cultures in black.

**Table S2. Dominant taxa presumptive amino acid fermenters compared to herb-selected taxa.** The most abundant taxa observed in control cultures that lack any supplemented carbohydrate source ferment amino acids for energy. Herb-supplemented cultures display an overall decreased relative abundance or nominal change compared to controls.

**Table S3.** **GH loci abundance.** Percent of microbiota encoding each GH loci (top). Differences in GH % abundance compared to control cultures (bottom).
